# Anticoagulation with warfarin and rivaroxaban ameliorates experimental autoimmune encephalomyelitis

**DOI:** 10.1186/s12974-017-0926-2

**Published:** 2017-07-28

**Authors:** Leonie Stolz, Amin Derouiche, Kavi Devraj, Frank Weber, Robert Brunkhorst, Christian Foerch

**Affiliations:** 1 Department of Neurology, Goethe University, Schleusenweg 2-16, 60528 Frankfurt am Main, Germany; 2 Dr. Senckenbergische Anatomie, Institute for Anatomy II, Goethe University, Frankfurt am Main, Germany; 3 Pharmazentrum Frankfurt, Institute for General Pharmacology and Toxicology, Goethe University, Frankfurt am Main, Germany; 4 Institute of Neurology (Edinger Institute), Goethe University, Frankfurt am Main, Germany; 5 Neurological Clinic Medical Park, Bad Camberg, Germany

**Keywords:** Multiple sclerosis, Experimental autoimmune encephalomyelitis, Inflammation, Mice, Anticoagulation

## Abstract

**Background:**

In multiple sclerosis, coagulation factors have been shown to modulate inflammation. In this translational study, we investigated whether long-term anticoagulation with warfarin or rivaroxaban has beneficial effects on the course of autoimmune experimental encephalomyelitis (EAE).

**Methods:**

Female SJL/J mice treated with anticoagulants namely warfarin or rivaroxaban were immunized with PLP_139–151_. Stable anticoagulation was maintained throughout the entire experiment. Mice without anticoagulation treated with the vehicle only were used as controls. The neurological deficit was recorded during the course of EAE, and histopathological analyses of inflammatory lesions were performed.

**Results:**

In preventive settings, both treatment with warfarin and rivaroxaban reduced the maximum EAE score as compared to the control group and led to a reduction of inflammatory lesions in the spinal cord. In contrast, therapeutic treatment with warfarin had no beneficial effects on the clinical course of EAE. Signs of intraparenchymal hemorrhage at the site of the inflammatory lesions were not observed.

**Conclusion:**

We developed long-term anticoagulation models that allowed exploring the course of EAE under warfarin and rivaroxaban treatment. We found a mild preventive effect of both warfarin and rivaroxaban on neurological deficits and local inflammation, indicating a modulation of the disease induction by anticoagulation.

**Electronic supplementary material:**

The online version of this article (doi:10.1186/s12974-017-0926-2) contains supplementary material, which is available to authorized users.

## Background

Multiple sclerosis (MS) is an autoimmune inflammatory disease characterized by demyelination, axonal damage, and gliosis. Platelets were shown to promote central nervous system (CNS) inflammation [1, 2], and proteins of the coagulation cascade were reported to accumulate in MS plaques [3]. In particular, thrombin and fibrin were proposed to be involved in MS pathophysiology [4, 5] by the modulation of local inflammatory responses, including microglial activation and plaque formation [6, 7]. Very recently, deficiency of the coagulation factor XII (the starting point of the intrinsic coagulation cascade) was shown to mitigate immune response in an experimental autoimmune encephalomyelitis (EAE) model [8]. In this explorative study, we investigated whether anticoagulation with the vitamin K antagonist warfarin or the factor Xa-inhibitor rivaroxaban has anti-inflammatory properties in EAE. For this purpose, we combined the EAE model in mice with more recently developed models of long-term anticoagulation. We examined the effects of anticoagulation on clinical endpoints as well as on histopathological parameters such as immune cell infiltration into the CNS.

## Methods

### Animals

One hundred seventy female SJL/J mice that are specific pathogen free and aged 9–10 weeks at immunization were purchased from Charles River (Sulzfeld, Germany) or Janvier (Le Genest-Saint-Isle, France) to study relapsing-remitting (RR-)EAE [9]. The animals were housed under a constant room temperature (23 ± 1 °C) and a 12 h light dark cycle (five mice per cage). Food and water were available ad libitum. This study was performed in strict compliance with the animal handling protocols approved by the Regierungspräsidium Darmstadt, Germany (Nos. FU1008, FU1098) and following the guide for the Care and Use of Laboratory Animals published by the National Institutes of Health. All the experiments complied with the ARRIVE guidelines for conducting animal experiments.

### Study design

We pre-defined the following experimental series: (i) preliminary studies in which we established the anticoagulation model in mice subjected to RR-EAE; (ii) randomized preventive treatment comparing the course of EAE under warfarin or rivaroxaban anticoagulation with mice having a normal coagulation status; and (iii) randomized therapeutic treatment comparing the course of EAE under warfarin anticoagulation with mice having a normal coagulation status. A pre-planned therapeutic treatment trial using rivaroxaban anticoagulation was not executed in view of the results obtained from the therapeutic warfarin study.

In the preventive treatment trial, the mice were randomized, and anticoagulation therapy was started 1 day before immunization. In the therapeutic treatment trial, the mice were allocated pairwise to control and anticoagulation treatment groups according to their clinical scores on day 12 post immunization, allowing an identical distribution of EAE scores in both groups at the onset of treatment [10].

### Sample size calculation

In both the preventive and the therapeutic randomized trials, we used a sample size of 20 mice per group. Sample size calculation was performed using an online calculation tool [11]. Aiming for a mean EAE score reduction of 25% (e.g., from an average of 2 to an average of 1.5) by treatment and assuming a standard deviation of 0.5 points of the EAE score, a sample size of 18 animals per group was necessary for a probability of an alpha-error of 0.05 and a power of 0.80 (two-sided test) applying the Mann-Whitney *U* test.

### Long-term anticoagulation regimen in EAE

In previous translational studies on anticoagulation in stroke, warfarin was applied via drinking water [12–14]. However, in a pilot trial, we found strong variations in daily water intake during the course of EAE, depending on the clinical status of the animals (about 4 ml prior to immunization vs. about 1.5 ml during the peak of EAE; see Additional file 1: Figure S1a). Consequently, we applied warfarin via subcutaneous (s.c.) injections. For doing so, a 5-mg coumadin tablet (warfarin sodium, crystalline, Bristol Myers Squibb, New York, USA) was dissolved in sterilized water. The mice were treated every 12 h starting with a warfarin dose of 0.50 mg/kg body weight followed by a maintenance dose of 0.13–0.18 mg/kg body weight (dose-finding experiments are shown in Additional file 1: Figure S1b, c). To monitor stable anticoagulation within an international normalized ratio (INR) target range of 2–4 during the experiment, the INR was measured every 12–24 h in three randomly selected mice via a point-of-care INR device (see below). Warfarin dosing was then adjusted if necessary. The mice in the control group were injected in a similar way (s.c., in 12 h intervals) with the solvent only, and INR was determined in three randomly selected mice every 12–24 h.

For long-term anticoagulation with rivaroxaban, a 20-mg Xarelto tablet (Bayer, Leverkusen, Germany) was dissolved in 3.3 ml tap water. A dose of 30 mg/kg body weight was administered every 12 h for 3 days per oral gavage as described previously [15]. In a pilot trial, we measured thrombin activity at 0, 1, 2, 3, and 4 h after rivaroxaban administration to evaluate the effects on the systemic coagulation. Furthermore, we determined the rivaroxaban plasma concentration at 1 h after the treatment. In the EAE experiment, we measured thrombin activity and rivaroxaban plasma concentration at the first peak (d15) and at the time point of study termination (second peak, d27). The target range of rivaroxaban plasma concentration was 150–250 ng/ml [16].

### Coagulation tests

#### INR

Eight microliters of whole blood was taken from the tail vein of the mice and applied to INR test stripes (CoaguChek, Roche, Basel, Switzerland). INR measurements using a point-of-care device have previously been established in rodents [17].

#### Rivaroxaban plasma concentration

A transcardial puncture was performed in anesthetized mice. Four hundred fifty microliters of whole blood was taken from the left ventricle in a 1-ml syringe coated with 50 μl citrate. The citrated blood was centrifuged for 15 min at 1500*g*. Rivaroxaban plasma concentrations were determined via mass spectroscopy (as previously described [18]).

#### Thrombin activity assay

One microliter of the obtained plasma was used to quantify the proteolytic activity of thrombin through measuring the cleavage of the synthetic fluorogenic peptide substrate Boc-Asp(OBzl)-Pro-Arg-AMC (Bachem, Bubendorf, Switzerland) [19]. For doing so, plasma was added to the substrate buffer which consists of 50 mM TRIS/HCl, pH = 8, 0.15 M NaCl, 1 mM CaCl_2_, 0.1% BSA, 0.1 mg/ml bestatin (Cayman Chemical Company, Ann Arbor, USA), and 0.2 mM prolyl endopeptidase inhibitor (Merck, Darmstadt, Germany) in a 96-well black microplate (Nunc, New York, USA). To start the reaction, 14 μM substrate was added to a final volume of 100 μl. Measurements were performed by the VICTOR3 Multilabel Counter (PerkinElmer, Waltham, USA) with excitation and emission filters of 360 ± 35 and 460 ± 35 nm. For calibration, a standard curve with known bovine thrombin concentrations (cat. no. T4648, Sigma-Aldrich, St. Louis, USA) was used and two wells containing only the buffer served as blank.

### EAE

EAE was induced by s.c. injections of 100 μg PLP_139–151_ in 100 μl complete Freund’s adjuvant at two sites of the SJL/J mice followed by two intraperitoneal injections of 400 ng pertussis toxin 2 and 24 h after PLP injection (Hooke Laboratories, Lawrence, USA).

The mice were weighted and scored by a blinded rater daily for clinical symptoms according to the following EAE score: 0, no clinical disease; 0.5, weak tail tip; 1, limp tail; 1.5, limp tail + one weak hindlimb; 2, limp tail + two weak hindlimbs; 2.5, limp tail + paralysis of one hindlimb; 3, limp tail + complete hindlimb paralysis; 3.5 limp tail + complete hindlimb paralysis + one weak forelimb; 4, limp tail + complete hindlimb paralysis + weak forelimbs; >4 moribund or dead [20]. At the first EAE peak (d15), three mice per group were sacrificed in the warfarin experiment for histological analysis. In the rivaroxaban experiment, five mice per group were sacrificed at the first peak for histological analysis and to monitor the anticoagulation status. The mean EAE score was calculated every day based on all available animals per group. The mean maximum EAE score was calculated based on the maximum scores of individual mouse during the experiment, except for the mice that died at the beginning of the experiment.

### Histopathological analysis

Immune cell infiltration was assessed via DAPI staining of the spinal cord. Histological samples were taken on d15 and d27 after immunization (first and second peak). After deep anesthesia with isoflurane, the mice were transcardially perfused with ice-cold 0.1 M phosphate buffer (6.4 mM NaH_2_PO_4_, 154 mM Na_2_HPO_4_, pH 7.4) followed by perfusion with 4% paraformaldehyde (20 min). The spinal cord was removed and post-fixed for 1.5 h in 4% paraformaldehyde followed by 8 h in 10%, 8 h in 20%, and 3 h in 30% sucrose for cryoprotection. Ten-micrometer sections were cut with a cryostat from three specific regions in the spinal cord (cervical, thoracic, and lumbar region). Unstained sections were inspected for signs of intraparenchymal hemorrhage. The sections were permeabilized with phosphate-buffered saline containing 0.1% Tween-20. To show inflammatory lesions, the slides were incubated for 10 min with DAPI (1:1000, Sigma-Aldrich, St. Louis, USA), washed and mounted with coverslips.

### Imaging and analysis

All stainings were examined by epifluorescence microscopy (Axio Imager.A2, Zeiss, Jena, Germany), at ×2.5 magnification. At least five sections per defined region of the spinal cord were examined from a blinded rater who performed the counting of DAPI-stained cell clusters. Each cluster was counted as one lesion.

### Isolation of porcine brain microvascular endothelial cells (PBMECs)

The endothelial cells were isolated from fresh brains as previously described [21] with minor modifications. In brief, the porcine brain was removed from the skull and the meninges were removed via forceps. The cerebrum was minced and homogenized in buffer A (153 mM NaCl, 5.6 mM KCl, 1.7 mM CaCl_2_, 1.2 mM MgCl_2_, 15 mM HEPES, 10 g/l BSA, pH 7.4) using a dounce homogenizer (0.025 mm clearance, Wheaton, Millville, USA) and centrifuged at 400*g* for 10 min at 4 °C. The pellet was resuspended and digested with 0.25% collagenase II (Worthington, Lakewood, USA) in buffer A for 1 h on a rocker at 37 °C. To remove myelin, the pellet was resuspended in 25% BSA/PBS and centrifuged at 2000*g* for 30 min at 4 °C, followed by further enzymatic digestion with 1 mg/ml collagenase/dispase (Roche, Basel, Switzerland) and 1 μg/ml DNase I (Worthington, Lakewood, USA) in buffer A for 15 min at 37 °C. The obtained PBMECs were resuspended in MCDB-131 complete medium [22] and seeded on T75 cell culture flasks pre-coated with type I collagen (150 μg/cm^2^, Corning, Corning, USA). After 12 h, the cells were cultured in 4 μg/ml puromycin (Sigma-Aldrich, St. Louis, USA) for 2 days, to select endothelial cells [21].

### Transendothelial electrical resistance (TEER) measurements

PBMECs (50,000 cells/cm^2^) were seeded onto 24-well PET transwell inserts (1-μm pore, Greiner Bio-One, Kremsmünster, Austria) pre-coated with fibronectin (5 μg/cm^2^, Sigma-Aldrich, St. Louis, USA). The inserts were transferred to a cellZscope device® (Nano Analytics, Münster, Germany) placed in a humidified incubator, and TEER values were obtained from continuous impedance measurements [22]. Thrombin treatment (0.5, 1, 10 U; Prospec, Ness Ziona, Israel) was started after TEER values reached a plateau. As a control, the cells were treated with the diluent of thrombin.

### Statistical analysis

Data were presented as mean ± SEM. Statistical analysis was performed using GraphPad Prism version 5.04 for Windows (GraphPad Software, San Diego, USA). The lesion burden of histological sections was compared using the *t* test. Data concerning the EAE score were compared using two-sided Mann-Whitney *U* test.

## Results

### Model of long-term anticoagulation in EAE mice

#### Warfarin anticoagulation

**Fig. 1:**
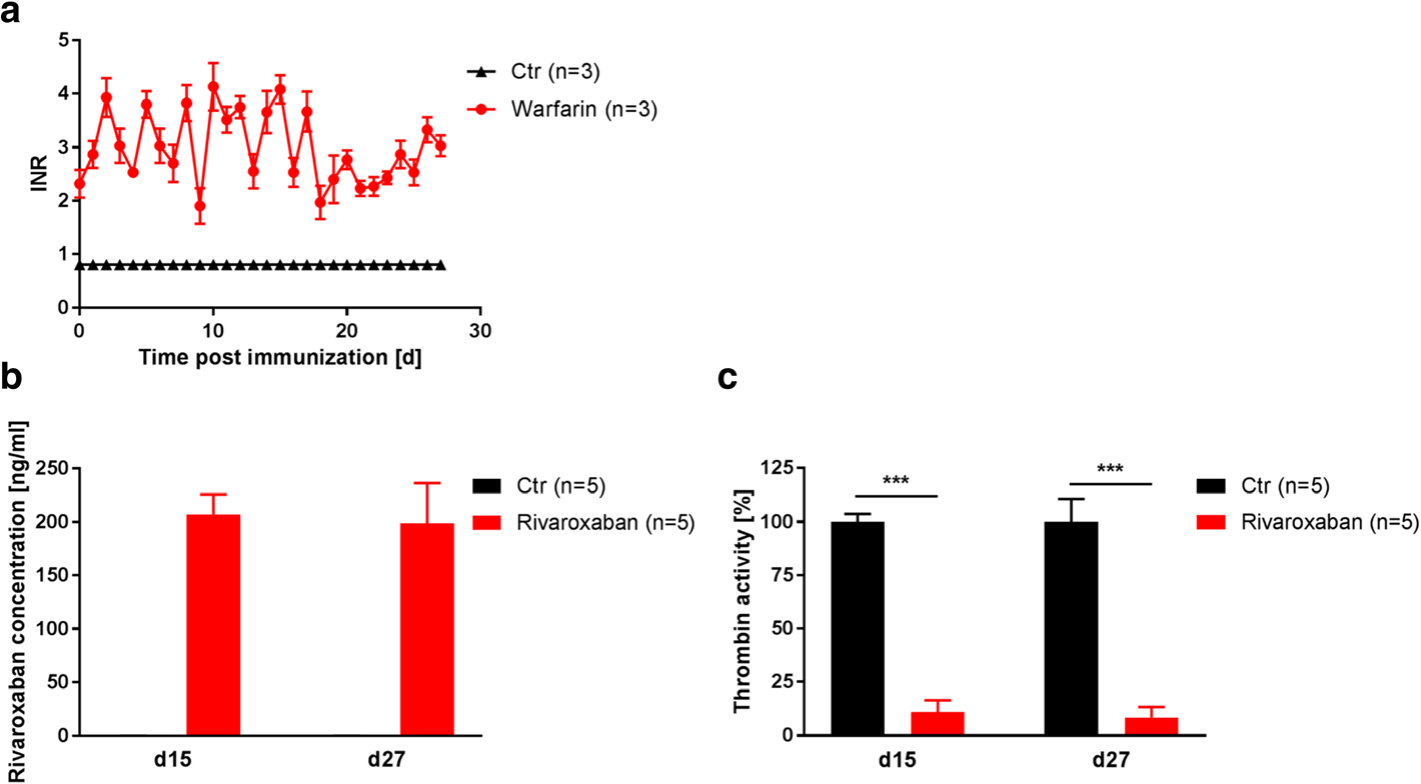
Long-term anticoagulation of SJL/J mice. PLP_139–151_-immunized mice were anticoagulated with warfarin via s.c. injections every 12 h during the course of the experiment. INR was measured on a regular basis (**a**). PLP_139–151_-immunized mice were anticoagulated with rivaroxaban. Rivaroxaban plasma concentration and thrombin activity in the plasma were measured on d15 and d27 post immunization (**b**, **c**)

Water consumption in the untreated mice was 4 ml per day. In the mice subjected to EAE, water consumption dropped to 1.5 ml per day at day 12 after immunization (Additional file 1: Figure S1a), making a drinking water-based application of warfarin impracticable. Instead, warfarin was applied subcutaneously with an initial dose of 0.5 mg/kg body weight. This was followed by maintenance doses of 0.13–0.18 mg/kg body weight via s.c. injections, depending on the actual INR values (see Additional file 1: Figure S1b, c for dose-finding experiments). By doing so, a stable anticoagulation with INR values between 2 and 4 could be maintained during the course of EAE (Fig. 1a). The control animals had INR values of 0.8 in all cases.

#### Rivaroxaban anticoagulation

In the pilot trial, a mean (±SEM) rivaroxaban plasma concentration of 218 ± 43 ng/ml was determined 1 h after the last administration. In the controls, rivaroxaban concentrations were (artificially) determined to be 0.3 ± 0.1 ng/ml (Additional file 2: Figure S2a). Rivaroxaban anticoagulation reduced thrombin activity in the pilot trial by 85 ± 5% (*p* < 0.001, Additional file 2: Figure S2b). The maximum thrombin inhibition was reached 1 h after rivaroxaban application. Four hours after the application, the thrombin activity was restored to the activity level before the treatment (Additional file 2: Figure S2c). In the randomized preventive trial, rivaroxaban plasma concentrations were 207 ± 16 ng/ml (d15) and 199 ± 34 ng/ml (d27), respectively, 1 h after the last application (Fig. 1b). Thrombin activity was reduced by 89 ± 5% (d15) and 91 ± 4% (d27), respectively, in the treated animals compared to the controls (*p* < 0.001 Fig. 1c).

### Effect on neurological symptoms

#### Preventive treatment with warfarin ameliorates the clinical course of EAE

**Fig. 2:**
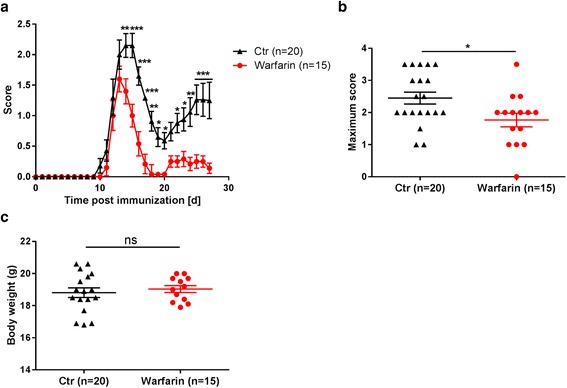
Preventive anticoagulation with warfarin ameliorates neurological deficits of EAE. Forty mice were immunized on d0 with PLP_139–151_ and 20 were treated with warfarin, starting on d1. The clinical scores of the mice were measured daily (**a**). Maximum scores were found decreased in warfarin-treated mice as compared to the control group (**b**). There was no difference in loss of weight at the end of the experiment between the groups (**c**)

In the warfarin group, five mice died between the time point of immunization and the development of the first clinical symptoms of EAE due to bleeding complications at the site of drug administration. These mice were excluded from all analyses. All other animals survived until the pre-planned end of the study. Compared to the control group lacking anticoagulants, the warfarin-treated mice showed a reduced mean EAE score, starting with the first peak of the disease (d15) until the end of the experiment on d27 (d15: control: 2.2 ± 0.2 vs. warfarin: 1.0 ± 0.2; *p* < 0.001; d27: control: 1.3 ± 0.1 vs. warfarin 0.1 ± 0.1; *p* < 0.001, Fig. 2a). There was a reduction of the maximum EAE score (control: 2.5 ± 0.2 vs. warfarin: 1.8 ± 0.2; *p* = 0.02, Fig. 2b), but no difference in loss of weight between groups (Fig. 2c).

#### Preventive treatment with rivaroxaban ameliorates the clinical course of EAE

**Fig. 3:**
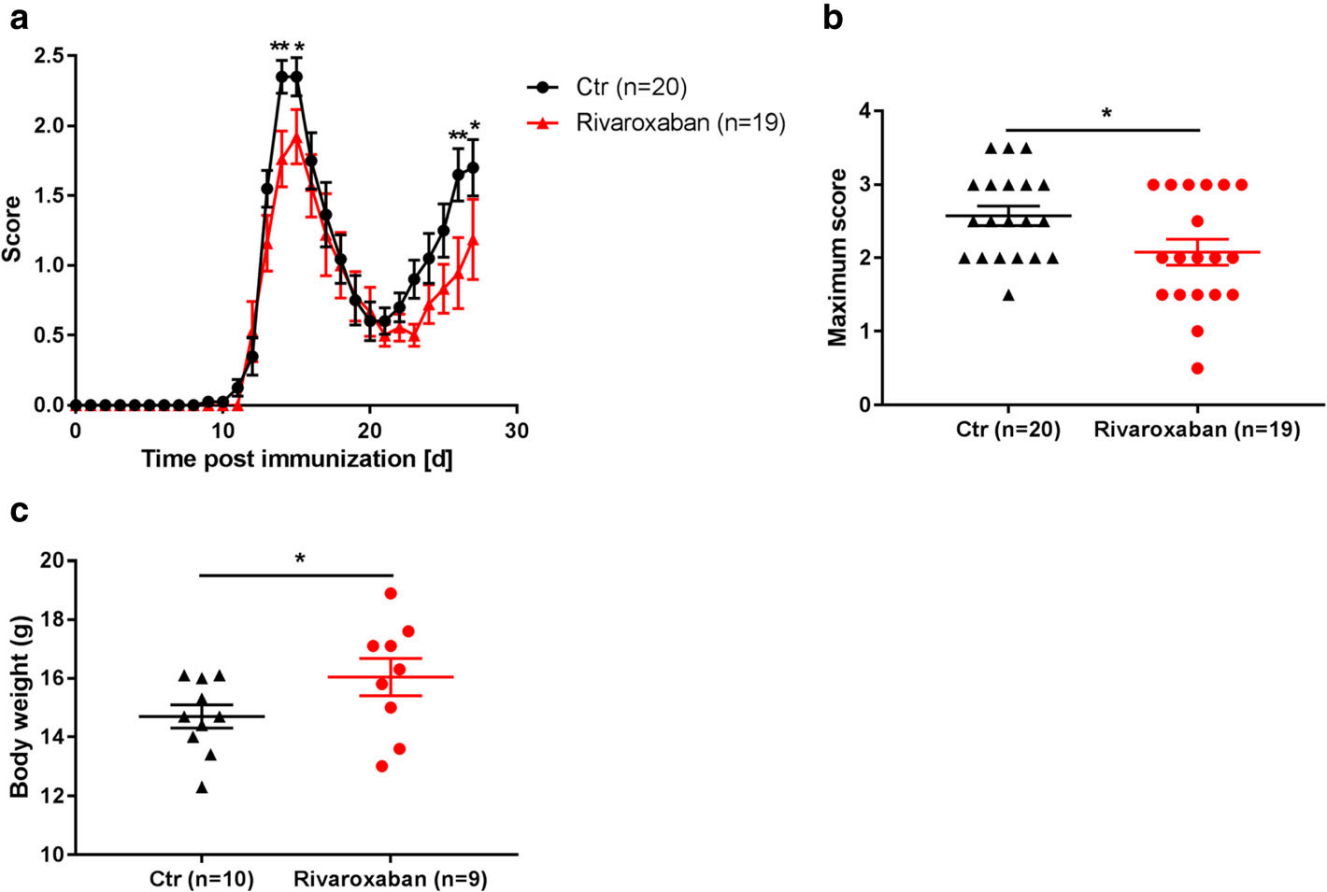
Preventive anticoagulation with rivaroxaban ameliorates neurological deficits of EAE. Forty mice were immunized on d0 with PLP_139–151_ and 20 were treated with rivaroxaban, starting on d1. The clinical score of the mice were measured daily (**a**). Maximum scores were found decreased in rivaroxaban-treated mice as compared to the control group (**b**). Loss of weight was reduced in the anticoagulated group at the end of the experiment (**c**)

In the rivaroxaban group, one mouse died because of bleeding complications at the time point of immunization. This mouse was excluded from all analyses. Compared to the control group, the rivaroxaban-treated animals showed a reduced mean EAE score at the first and second peaks of the disease (d15: control: 2.4 ± 0.1 vs. rivaroxaban 1.8 ± 0.2; *p* = 0.04; d27: control: 1.7 ± 0.1 vs. rivaroxaban 1.2 ± 0.2; *p* = 0.01, Fig. 3a). Rivaroxaban treatment also led to a decrease of the maximum EAE score (control: 2.6 ± 0.1 vs. rivaroxaban 2.1 ± 0.2; *p* = 0.03) as well as to a reduced loss of weight at the point of study termination (control: 14.7 ± 0.4 g vs. rivaroxaban: 16.1 ± 0.6; *p* = 0.04, Fig. 3b, c).

#### Therapeutic treatment with warfarin does not affect the course of EAE

In the warfarin group, two mice died during the treatment regime due to bleeding complications and one mouse died in the control group after EAE induction. These mice were excluded from all analyses. There was no difference in the clinical score as well as in the body weight of the treated mice compared to the control group (see Additional file 3: Figure S3).

### Histopathological analysis

#### Preventive treatment with warfarin and rivaroxaban reduces inflammatory infiltrates in the spinal cord

**Fig. 4:**
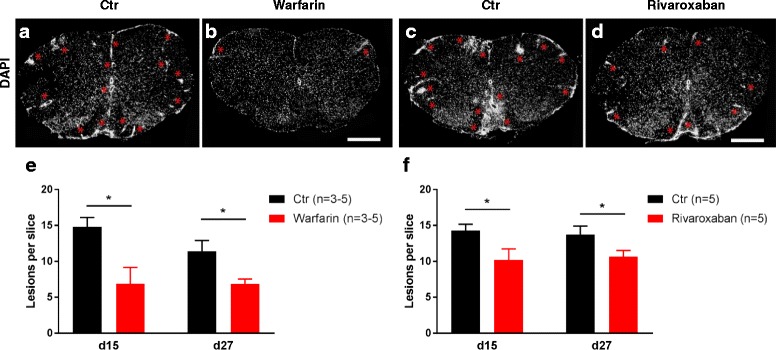
Preventive anticoagulation with warfarin and rivaroxaban decrease inflammatory lesions in the spinal cord of PLP_139–151_-immunized SJL/J mice. Representative images of DAPI stained lumbar spinal cord sections for the respective groups. Inflammatory lesions are marked by *asterisk* (**a**–**d**, ×2.5 magnification, scale bar = 500 μm). Quantitative analysis of the cervical, thoracic, and lumbar spinal cord shows reduced inflammatory lesions on d15 and d27 after immunization in anticoagulated mice compared to controls (**e**, **f**)

Warfarin and rivaroxaban treatment led to a reduction of inflammatory lesions (assessed via DAPI staining, Fig. 4a–d) in the spinal cord of the EAE mice compared to the control group. Quantitative analysis of the spinal cord showed a significant reduction of the lesion burden in the first and second peaks of the disease (d15: warfarin: 14.8 ± 1.1 vs. 6.9 ± 1.9 [*n* = 3] lesions per slice; *p* = 0.04; rivaroxaban: 14.3 ± 1.4 vs. 10.2 ± 0.8 [*n* = 5] lesions per slice; *p* = 0.04; d27: warfarin: 12.3 ± 1.3 vs. 6.7 ± 0.3 [*n* = 5] lesions per slice; *p* = 0.03; rivaroxaban: 13.4 ± 1.0 vs. 10.6 ± 0.7 [*n* = 5] lesions per slice; *p* = 0.04; see Fig. 4e, f). There were no signs of intraparenchymal hemorrhage in any of the groups.

### TEER measurement

#### Blood-brain barrier (BBB) opening effects of thrombin

**Fig. 5:**
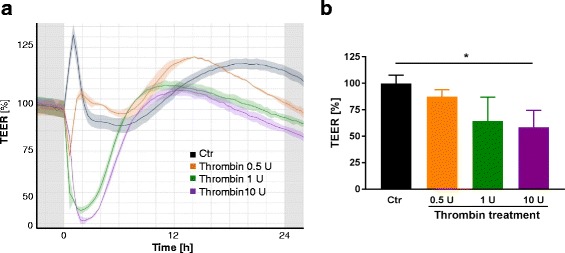
Thrombin increases brain endothelial permeability in vitro. PBMECs were seeded on transwell inserts and continuous TEER values were measured by a cellZscope® system. The graph displays a representative experiment that shows reduced TEER values of thrombin-treated PBMECs compared to control-treated ones (**a**). Quantification of brain endothelial permeability 1 h after thrombin treatment shows a decrease of TEER values in a dose-dependent manner. At least three replicates were averaged for one biological sample (*n* = 3, **b**)

Warfarin and rivaroxaban treatment led to an inhibition of the coagulation protease thrombin. Since thrombin is known to have effects on the permeability of endothelial cells, we analyzed the effects of thrombin on BBB integrity by in vitro TEER measurements. Freshly isolated PBMECs were treated with different doses of thrombin (0.5, 1, and 10 U), and the endothelial permeability was measured over 24 h (Fig. 5a). The TEER values decreased after the treatment in a dose-dependent manner, indicating a barrier opening effect of thrombin. The lowest values were reached 1 h after the treatment, reducing the measured resistance by 13–41% (0.5–10 U, Fig. 5b).

## Discussion

Our study was designed to examine the anti-inflammatory potential of anticoagulation with warfarin and rivaroxaban in an experimental model of RR-MS. We identified a moderate protective effect for both substances if given in a preventive regimen (i.e., before immunization). In contrast, despite sufficient anticoagulation, therapeutic treatment with warfarin (i.e., starting after EAE establishment) had no effect on the EAE course.

Rodent models of anticoagulation have recently been developed in the context of stroke and traumatic brain injury [12, 15, 23, 24]. However, such models have not been applied to the mice subjected to EAE. For this purpose, we had to modify the anticoagulation regime utilized in previous studies [12, 15] in order to maintain anticoagulation in neurologically impaired mice for several weeks. We showed that subcutaneous administration of warfarin (instead of applying it via drinking water as reported previously [12, 13, 25]) resulted in fairly stable anticoagulation within the target range (INR 2–4) during the entire phase of EAE. In the rivaroxaban treatment groups, we obtained rivaroxaban plasma concentrations that were comparable to those measured in rivaroxaban-exposed humans [16]. Despite uncertainties regarding the “true” anticoagulant effect of the medication in the mice, we consider the vast reduction of thrombin activity as an indicator of effectiveness [26].

A preventive and a therapeutic treatment paradigm were tested in the RR-EAE model. The severity of EAE (in terms of ordinal EAE scores) was reduced by both warfarin and rivaroxaban anticoagulation in the preventive trial. The warfarin treatment let to a significant reduction of the EAE scores starting at the first peak of the disease until the end of the experiment. In contrast, rivaroxaban treatment led to a reduction of the EAE scores during the first and second peaks of the disease only. One reason for the stronger effect of warfarin on the EAE scores compared to rivaroxaban might be the shorter half-life of rivaroxaban. Whereas warfarin treatment led to a sustained anticoagulation during the whole EAE experiment, rivaroxaban influenced the coagulation system in a more “pulsating” way (as in humans [27]), where thrombin activity was already restored 4 h after the rivaroxaban gavage. Histopathological analyses showed reduced inflammatory infiltrates in the mice treated with the anticoagulants compared to the controls. However, effect size of the preventive treatment on the EAE score and on the histopathology appeared to be smaller in comparison to drugs in clinical use [28, 29]. We observed a reduction in weight loss at the end of the study period in the rivaroxaban-treated mice but not in the warfarin-treated animals. In the warfarin experiments, we injected the animals twice per day with warfarin or the solvent, respectively, and took blood from three mice per group each day. We hypothesize that these stress conditions, together with the EAE, led to a reduction of body weight in both groups [30, 31]. In contrast, in the rivaroxaban experiments, we applied the treatment orally and collected blood only when sacrificing the animals (d15, d27), leading to reduced stress conditions.

When warfarin was used as a therapeutic agent, we did not observe a difference in EAE severity between the warfarin-treated mice and the control mice with a normal coagulation system. Since the effects in preventive treatment regimens are always bigger than those in therapeutic ones [10, 32], the already small effect in the preventive warfarin experiment could explain the lack of effects in the therapeutic setting. Another explanation is an influence of the anticoagulation on the pathophysiological mechanisms during the induction phase of EAE until the first peak and not on the mechanisms responsible for the later relapse and remission phase [33]. An early step in the induction phase is the loss of BBB integrity due to morphological changes of endothelial cells [34]. The finding that both warfarin and rivaroxaban had preventive effects in EAE endorses that downstream proteins (such as thrombin and fibrin) of the coagulation cascade are of importance in this context. Since thrombin is known to interrupt endothelial barrier integrity through PAR-1 signaling [35], the protective effect in the preventive setting might be explained by preserving the BBB integrity due to low thrombin activity under anticoagulation. Indeed, in our TEER experiments, we confirmed a BBB opening effect of thrombin. However, coagulation proteins from the extrinsic pathway (including factor VII) are inhibited by warfarin only (and not by rivaroxaban) and cannot be solely responsible for our findings (but could be a reason for the stronger effect of warfarin treatment compared to rivaroxaban in the amelioration of the disease course). In earlier studies, the inhibition of the coagulation factors thrombin and fibrin with hirudin or ancrod showed amelioration of EAE also in a therapeutic setting [3, 36]. These effects have been shown based on the disruption of the fibrinogen-microglia interaction via the CD11b receptor, leading to microglia activation [36]. In our study, we could not see a similar positive effect of therapeutic anticoagulation. One explanation might be the different targets of warfarin compared to hirudin and ancrod. Whereas the vitamin K inhibitor warfarin acts on proteins from the extrinsic and intrinsic coagulation cascade, hirudin and ancrod directly inhibit thrombin and lyse fibrin clots. The additional inhibition of anti-inflammatory coagulation factors like APC [37] by warfarin might reverse its positive effect on thrombin inhibition and possibly other coagulation factors.

In the current study, we have developed a model of long-term anticoagulation, sustainable during the course of EAE. This model might be used in future translational studies, whenever the course of the disease could interfere with the uptake of anticoagulants via drinking water. A potential weakness is that five mice died in the warfarin group due to bleeding complications after EAE induction. Since the animals already died during the first days after immunization (prior to first symptoms), it is unlikely that the differences in EAE scores are simply a result of a selection bias (i.e., a better outcome in the warfarin group due to the survival of less affected mice). Despite careful handling, bleeding complications are not always preventable in long-term anticoagulation therapies.

## Conclusion

In summary, we found a mild preventive effect of anticoagulation, induced either by warfarin or rivaroxaban, on neurological deficits and inflammation in EAE. Future studies are needed to clarify from a clinical perspective whether anticoagulants have a meaningful benefit in autoimmune CNS disease.

## Additional files

Additional file 1: Figure S1. Warfarin dose-finding experiments. For oral anticoagulation with warfarin, the water consumption of healthy SJL/J mice was measured at least every other day. The water consumption strongly declined after PLP_139–151_ injections (a). For anticoagulation via s.c. injections, three different loading doses of warfarin were tested in SJL/J mice (*n* = 3 per group) and the INR was measured after 12 h. A concentration of 0.5 mg/kg body weight leads to an INR in the therapeutic range (2.6 ± 0.2, b). Maintenance doses of 0.1–0.23 mg/kg body weight were tested after the application of 0.5 mg/kg body weight. Doses between 0.13 and 0.18 mg/kg body weight keep the INR almost stable (*n* = 3 per group, 2.1 ± 0.1–2.6 ± 0.6, c). (DOC 95 kb)

Additional file 2: Figure S2. Effects of rivaroxaban on the coagulation status of SJL/J mice. For anticoagulation with rivaroxaban, SJL/J mice were treated with 30 mg/kg rivaroxaban for 3 days every 12 h via oral gavage. One hour after the last application, the rivaroxaban plasma concentration (a) and the thrombin activity (b) were determined in treated animals and controls (*n* = 5 per group). To monitor the thrombin activity over time, 0, 1, 2, 3, and 4 h after the application, the thrombin activity was measured (c, *n* = 5 per time point). (DOC 68 kb)

Additional file 3: Figure S3. Therapeutic anticoagulation of PLP_139–151_-immunized SJL/J mice with warfarin. Forty mice were immunized on d0 with PLP_139–151_ and 20 were treated with warfarin, starting on d12. The clinical score of the mice was measured every day, and the weight of the mice was depicted at the end of the experiment (a, b). (DOC 76 kb)

## Abbreviations

BBB: Blood-brain barrier
CNS: Central nervous system
EAE: Experimental autoimmune encephalomyelitis
INR: International normalized ratio
MS: Multiple sclerosis
PBMEC: Porcine brain microvascular endothelial cells
PLP: Proteolipid protein
RR: Relapsing-remitting
S.c.: Subcutaneous
TEER: Transendothelial electrical resistance
