# Supplementary material for: Anticoagulation with warfarin and rivaroxaban ameliorates experimental autoimmune encephalomyelitis
Source: J Neuroinflammation. 2017 Jul 28;14:152. doi: 10.1186/s12974-017-0926-2 (PMC5534067; doi:10.1186/s12974-017-0926-2)
Supplement: Supplementary file 3 — Therapeutic anticoagulation of PLP139–151-immunized SJL/J mice with warfarin. Forty mice were immunized on d0 with PLP139–151 and 20 were treated with warfarin, starting on d12. The clinical score of the mice was measured every day, and the weight of the mice was depicted at the end of the experiment (a, b). (DOC 76 kb) [file 12974_2017_926_MOESM3_ESM.doc]

Additional file 3: Figure S3
